# Social Media Reinvented: Can Social Media Help Tackle the Post-Pandemic Mental Health Onslaught?

**DOI:** 10.7759/cureus.21070

**Published:** 2022-01-10

**Authors:** Ashish Sarangi, Wail Amor, Edzel Lorraine F Co, Sana Javed, Sadia Usmani, Aimn Rashid

**Affiliations:** 1 Geriatric Psychiatry, Baylor College of Medicine, Houston, USA; 2 Psychiatry, Texas Tech University Health Sciences Center, Lubbock, USA; 3 Psychiatry, University of Santo Tomas Faculty of Medicine and Surgery, Manila, PHL; 4 Psychiatry, Nishtar Medical University, Multan, PAK; 5 Psychiatry, Dow University of Health Sciences, Karachi, PAK

**Keywords:** mental health, covid-19, electronic media, pandemic, social media

## Abstract

Social media plays an omnipresent role in our modern lives, influencing every aspect of it, mental health being one of them. There has been increased focus on social media in recent times, especially its role in the ongoing coronavirus disease 2019 (COVID-19) pandemic. With various forms of social media, we hope to review the impact of various online platforms and emphasize their usage during the novel COVID-19 pandemic, which has led to a universal feeling of consternation throughout the world, mainly as a consequence of social distancing norms and cancellations of schools and places of work, which has affected not only the livelihood but also the way of life of scores of people.

Despite the negative press social media often receives in the field of mental health, there are opportunities to utilize its impact positively. This is not limited to connecting persons to mental health resources, spreading information about available COVID-19 treatment, and allowing social connection across the world. In this article, we review the renewed role that social media has had and future possibilities for its usage in the fight against the current pandemic.

## Introduction and background

The coronavirus disease 2019 (COVID-19) pandemic has had direct and indirect effects on the mental health of individuals worldwide [1]. This is the first pandemic where social media platforms have been the main source of information for the general population. Gao et al. and Moghanibashi-Mansourieh reported that anxiety and stress increased due to exposure to news and information on social media [1,2].

With multiple countries taking up strict lockdown measures, social media platforms have become the only source of human interaction and information about the pandemic, aiding people in maintaining connectivity. Social media has become an “influential” factor in the daily lives of many people; up to 50.64% of the 7.77 billion people in the world use social media [2]. Because of the rapidity of information dissemination from one part of the world across the other side using Facebook, Instagram, Twitter, and other online news sites, social media platforms have been utilized for decision-making on health matters by a majority of people.

Social media has been a boon and a bane in these tough times. It has been the major source of news about COVID-19, updating people about daily cases and new advancements in COVID-19 management. However, it is also inevitable that the spread of wrong information and other rumors has flooded the social media newsfeed. Lots of confusion and stress resulted from this; therefore, every single detail of the post should be screened and monitored.

## Review

We conducted a thorough database search of common databases (i.e., PubMed, Scopus, EBSCO, and Google Scholar) using the search terms “social media,” “pandemic,” “online platforms,” and “COVID-19” in various combinations. We included studies conducted in 2020, followed by a detailed abstract review. Book reviews, commentaries, and editorials were excluded. The initial search revealed a total of 214 articles, and after the exclusion, 31 articles were finalized and used for this narrative review. Additional studies were identified by examining the reference lists of the searched articles. For comprehensiveness, especially regarding the nonpsychiatric components of our review, we sourced literature from medical and basic science journals.

Information from the obtained articles was used to gauge the various effects of social media on the mental health of the population at large, weighing both the advantages and disadvantages (risks) of social media usage during the ongoing novel COVID-19 pandemic.

Elaborating mental health and its disruption by COVID-19

The World Health Organization (WHO) defines mental health as “a state of well-being in which every individual realizes his or her potential, can cope with the normal stresses of life, can work productively and fruitfully, and can make a contribution to her or his community.” The novel COVID-19 pandemic along with the unprecedented mitigation efforts that have disrupted the daily lives of millions has wreaked havoc on every aspect of mental health for the population worldwide mostly by enforcing unwanted isolation and limiting free movement. The ongoing pandemic has led to a chaotic world with a huge impact on the state of mind of humanity. The WHO together with other international health agencies has acknowledged some circulating threats to pandemic preparedness and control of people. This is especially true now with the circulating omicron variant, which is deemed to be more transmissible. The threats have included COVID-19 stigma, rumors, and other infodemics, a term that was coined way back in 2019 during the Ebola outbreak to mean a mixture of accurate and exaggerated information, something that makes it hard to decipher which one is trustworthy or not [3]. Because of this turmoil, people had gone panic-buying of essential items, which had led to supply shortages and price inflation. Managing these global enemies toward health is a great challenge to health agencies.

Using devices more, news, and comparison

The population has resorted to personal electronic gadgets more often to keep abreast with news and other information about the pandemic and, at the same time, to have some time to distract themselves against the constant influx of stress from what they get from browsing over the Internet. As they passively scroll through their social media applications such as Instagram and Facebook, they tend to have feelings of inadequacy, envy, and insecurity [4]. This is detrimental to mental health as it could lead to psychiatric illnesses such as depression and anxiety [5].

Advertising with a focus on substance abuse

Advertising is an important aspect of social media usage and has been one of the trending topics in the recent past. Unfortunately, it has a long-term repercussion on our behavior, often arousing interest in certain products or behaviors. One such category that is a cause of great concern is alcohol product placement. Since the beginning of this decade, major alcohol companies have been advertising more and more through social media, as indicated by the increased engagement for alcohol brands on Facebook and Twitter. This method of marketing has been effective as a means to reach their target audience. As of 2015, there is already 30% of young Australian adults noticing promotional alcohol posts on social media. Emerging evidence suggests that exposure to conventional forms of alcohol advertising is associated with not only increased consumption but also early onset of drinking [4]. It attracts the younger ones to embrace the alcoholic lifestyle and could destroy their future if not corrected, and since alcohol intake causes serious harmful effects to one’s health, it could dampen the immune system and could potentially contract COVID-19 and even lead to death.

Cyberbullying and mental health

One of the biggest risks that social media users, particularly adolescents, face during the pandemic is cyberbullying. With social interaction being limited to the online realm brought about by lockdown, young adults have become more exposed to adversities than ever, ranging from lowered self-esteem to behavioral and emotional problems [6]. Coping strategies are needed to support the mental health of these children, most especially during the COVID-19 pandemic. The stigmata that they get from contracting COVID-19 can also be another cause of bullying. This double impact of problems on these children could worsen more if social media cyberbullying could not be controlled [7].

Benefits of social media

The use of social media applications can bring about the sense of feeling connected from virtual interaction with loved ones, thereby bridging the gap of distant physical isolation. This reduces loneliness, anxiety, and depression [8].

Maintaining connectivity

Social media sites have played a vital role in keeping us in touch with our loved ones through video call applications such as Duo, FaceTime, and WhatsApp. This has served an integral role in tidying us through lockdowns and enabling various multinational companies to keep their work going with their employees via video call conferences. Not only do our homes and offices embrace these promising features of social media, but even the nursing homes also promote the usage of social media to ensure that their residents can stay connected with their families and the rest of the world amidst crisis.

The management and staff of nursing centers have had to adapt to the “new normal” almost overnight for the sake of the elderly in their care. Several hospitals have been encouraging the usage of social media applications such as Facebook (or the use of video applications) for their patients so they can keep in touch with their families, as it might be the last time that they could see their relatives [9]. This has been possible with the relaxation of Health Insurance Portability and Accountability Act (HIPAA) laws and waivers from the Centers for Medicare & Medicaid Services (CMS). Since it is also difficult for other outpatients to consult with their doctors, telemedicine has been part of the new norm to provide remote access to health services, most especially to maintain mental sanity. Until the pandemic happened in 2020, telemedicine service was underutilized. In this COVID-19 era, telemedicine has been the backbone of consultations worldwide. Several doctors provide free consultations over the Internet, as well as mental health education for everyone [10].

Mental health

The ease of connectivity to the Internet has aided in providing people with platforms to address issues about mental health, paving an easier means of sharing one’s experiences on having gone through similar sentiments, thereby having a sense of security and comfort. This most especially applies to the healthcare professionals who are at greatest risk in this discombobulating situation [11].

On content analysis of Facebook, Twitter, and LinkedIn public comments, more than half were negative, and the commonest emotion expressed was frustration [12]. The top users of social media were noted to be those who have anxiety levels, but it is still unclear if anxiety led to increased use of social media or vice versa [13]. Social media users have been trying to cope up with loneliness by trying to vent out their emotions on these platforms, as a part of their psychological crises. Social isolation, discrimination, boredom, depression, and anxiety from financial losses brought about by business closures are certain examples of psychological stressors faced by many during the pandemic. The peak of social media volume has risen during the stay-at-home protocol, and social media use has been a coping mechanism to withstand the feelings of isolation [14]. The definite increase of social media usage during pandemics can thus be leveraged for formulating targeted campaigns.

Keeping up with information about the pandemic

With this crisis happening globally, communication is imperative to advise people on what they can and cannot do and keep them informed about the statistics of the pandemic, the number of people infected, the death toll, the recovery rate, high-risk areas or COVID-19 hotspots, and various measures that the government is taking. Social media has become a smooth and accessible source of information for keeping people on their toes during the pandemic.

Helping each other during COVID-19

Several social media sites have played a key role in mobilizing people to take the necessary action to protect themselves against COVID-19. Several groups on Facebook have been created to organize vaccination campaigns and help in rendering vaccinations by securing slots for those individuals from different walks of life. In these unprecedented times, camaraderie has been observed as several community pantries have been opened up for those hungry and suffering. Because the spread of information is so fast with the aid of social media, the good out of this community pantry done by just a certain community has been imitated rampantly by many communities all over the world [15,16].

Communication and logistics

In April 2021, India witnessed the second wave of the COVID-19 pandemic, and with the healthcare system stretched to the limit, the citizens in a display of solidarity have taken it upon themselves to fill the lack of a centralized or accurate source of information. One way that they have decided to do this is to take advantage of the various social media platforms available to them and share information about the various portals where they can gain information about the availability of beds, ventilators, and vaccination slots. Not only the public in general but also the doctors have their groups for discussing new developments, treatments, diagnoses, vaccination slots, and resources on applications such as WhatsApp and Telegram. Many developers also came up with applications and websites such as CovRelief; it tracks the availability of hospital beds, lists oxygen suppliers, shares videos from doctors, and has an updated list of state helpline numbers in real time [17,18].

Promoting vaccinations

Even before the pandemic hit, social media websites such as Facebook and Twitter were already proficient tools in spreading information about the benefits of vaccinations, blood donation drives, and free health campaigns to the public. This is usually more prevalent in developed countries such as Australia, where multiple vaccine-promoting organizations had utilized various social media applications because of the advantages and efficiency. Because the scope of advertising with social media is so huge, it is a great means of reaching out to people of various demographics to stimulate conversations around the topics of why we should get COVID-19 vaccines and address all misconceptions regarding the vaccine [19]. In line with the pandemic, the WHO, in collaboration with other government health offices across the world, has taken it upon itself to not only spread awareness about COVID-19 prevention but also tackle the “infodemic of false news about the virus and its treatment” using social media. Instagram has incorporated a feature that allows users to share a link to WHO’s information center (https://www.instagram.com/coronavirus_info), where they can provide more information about COVID-19 vaccination. Instagram has also allowed the spread of awareness by posting stories using built-in stickers related to the pandemic [8].

With the Internet being full of “pandemic conspiracy” theories and rumors, it is an absolute necessity that health organizations all over the world unite to put consistent effort to monitor and screen out and disseminate relevant information about COVID-19.

Support for people with disabilities

Social media platforms also address and cater to the special needs of individuals with disabilities since they are more vulnerable to face difficulties during disasters. As a way to respond to the emergency needs of this group of people, several special technological features have been added to social media applications, such as voice commands, screen readers, speech-to-text translators, eye gaze trackers, hearing aid tools, and other accessibility features. This will not only help people with disabilities with their daily basic needs but also avoid discrimination of being deprived of technological wonders [6,20].

Table 1 provides a summary of the advantages and disadvantages of social media during the COVID-19 pandemic.

**Table 1:** Advantages and disadvantages of social media during the COVID-19 pandemic.

	Remarks
Advantages	Maintains connectivity among people; facilitates continuous consultation with physicians through telemedicine; reduces loneliness, anxiety, and depression; updates everyone with the latest information about COVID-19; serves as an alternative platform to help each other during the pandemic; allows solidarity to combat COVID-19; caters to the needs of people with disability
Disadvantages	Gives a sense of insecurity to oneself and leads to psychiatric illnesses; promotes advertisement of the use of illegal substances; attracts cyberbullying, which can give a negative impact on adolescents

## Conclusions

Several factors can play into consideration when it comes to analyzing what social media can do to people when they try to embrace the new normal of pandemics. With social media providing such a great impact on our everyday lives, from being a major stressor to a source of relief, it is very hard to categorize it as either beneficial or detrimental. It is imperative to guide the public with its use accordingly to promote mental health awareness.
